# Simulated Microgravity Attenuates Stretch Sensitivity of Mechanically Gated Channels in Rat Ventricular Myocytes

**DOI:** 10.3390/ijms26146653

**Published:** 2025-07-11

**Authors:** Andrey S. Bilichenko, Alexandra D. Zolotareva, Olga V. Kamkina, Valentin I. Zolotarev, Anastasia S. Rodina, Viktor E. Kazansky, Vadim M. Mitrokhin, Mitko I. Mladenov, Andre G. Kamkin

**Affiliations:** Institute of Physiology, Pirogov Russian National Research Medical University, 117513 Moscow, Russia; bilichenko_as@rsmu.ru (A.S.B.); zolotareva_ad@rsmu.ru (A.D.Z.); kamkina_ov@rsmu.ru (O.V.K.); zolotarev_vi@rsmu.ru (V.I.Z.); rodina_as@rsmu.ru (A.S.R.); kazanskii_ve@rsmu.ru (V.E.K.); mitrokhin_vm@rsmu.ru (V.M.M.); andrey.kamkin@rsmu.ru (A.G.K.)

**Keywords:** mechanosensitive channels, simulated microgravity, stretch, TRPM7, rat, ventricular myocytes

## Abstract

Cardiomyocytes, similarly to cells in various tissues, are responsive to mechanical stress of all types, which is reflected in the significant alterations to their electrophysiological characteristics. This phenomenon, known as mechanoelectric feedback, is based on the work of mechanically gated channels (MGCs) and mechano-sensitive channels (MSCs). Since microgravity (MG) in space, as well as simulated microgravity (SMG), changes the morphological and physiological properties of the heart, it was assumed that this result would be associated with a change in the expression of genes encoding MGCs and MSCs, leading to a change in the synthesis of channel proteins and, ultimately, a change in channel currents during cell stretching. In isolated ventricular cardiomyocytes of rats exposed to SMG for 14 days, the amount of MGCs and MSCs gene transcripts was studied using the RNA sequencing method by normalizing the amount of “raw” reads using the Transcripts Per Kilobase Million (TPM) method. Changes in the level of channel protein, using the example of the MGCs TRPM7, were assessed by the Western blot method, and changes in membrane ion currents in the control and during cardiomyocyte stretching were assessed by the patch-clamp method in the whole-cell configuration. The data obtained demonstrate that SMG results in a multidirectional change in the expression of genes encoding various MGCs and MSCs. At the same time, a decrease in the TPM of the MGCs TRPM7 gene leads to a decrease in the amount of TRPM7 protein. The resulting redistribution in the synthesis of most channel proteins leads to a marked decrease in the sensitivity of the current through MGCs to cell stretching and, ultimately, to a change in the functioning of the heart.

## 1. Introduction

Cardiomyocytes, like many other cell types, are exquisitely sensitive to mechanical forces. Such forces significantly affect their electrophysiological performance. This phenomenon, termed mechanoelectrical feedback (MEF), plays a crucial role not only in maintaining normal cardiac physiology but also in the development of various cardiac pathologies, including arrhythmias [1]. In 1988, Craelius, Chen, and El-Sherif published work that laid the foundation for our understanding of stretch-activated channels (SACs) [2]. They were identified as essential mediators of Mechano-Electrical Feedback (MEF), inducing changes in membrane conductance in response to mechanical deformations. Patch-clamp techniques were used to identify ionic currents in neonatal rat ventricular myocytes triggered by membrane stretch induced via negative pressure. Subsequent studies have shown that the mechanical stretch of both atrial [3] and ventricular [4] cardiac tissue modulates both the resting membrane potential and action potential configuration. Similarly, stretch-induced activation of non-selective cation currents through mechanically gated channels (MGCs) has been observed in both ventricular [5,6] and atrial cardiomyocytes [7].

To classify ion channels that are sensitive to mechanical forces, we adopt a pragmatic distinction used in previous studies. Channels that are activated directly by mechanical stimuli, including stretch or shear transmitted through the cortical cytoskeleton, and hence act as mechanoelectrical transducers, are all classified as mechanically gated channels (MGCs) [8,9,10]. These are generally non-selective cation channels, although some are, to some extent, selective for potassium. On the other hand, there are mechanosensitive channels (MSCs), which consist of individually regulated voltage- or ligand-gated channels (e.g., Na_V_, Ca_V_, K_V_) that exhibit ion conductance modulations in response to mechanical tension, but are not directly gated by it [11,12,13,14]. For instance, mechanical stretch can suppress Ca^2+^ influx through L-type Ca_V_1.2 channels, highlighting their mechanosensitive nature [12].

Mechanical forces are present in any multicellular organism, and gravity is an inescapable source of such stress. The heart, in particular, is adapted to operate under the Earth’s gravitational load (1 g). Changes in gravitational conditions (spaceflight or simulated microgravity (SMG)) significantly affect the structure and function of the cardiovascular system [15,16]. These include reduced peripheral vascular resistance under microgravity [17] and SMG conditions [18], alterations in cardiac dimensions [19,20,21], shifts in cardiac output during and after spaceflight [22,23,24], and a reduction in heart mass and ventricular volume after prolonged unloading [25]. These physiological changes are often accompanied by an increased incidence of cardiac arrhythmias [26].

At the cellular level, microgravity and SMG can change the structure of cardiomyocytes and decrease the cross-sectional area, sarcomere length, mitochondrial content, and myosin ATPase activity [27,28]. The upregulation of calcium oscillation and altered expression of contractile proteins, including myosin heavy chains, has also been documented [29]. Furthermore, SMG induces nitric oxide (NO) synthesis and upregulates inducible NOS (iNOS) gene expression in cardiomyocytes [30].

At the cellular level, microgravity and SMG induce the structural remodeling of cardiomyocytes, including reductions in cross-sectional area, sarcomere length, mitochondrial content, and myosin ATPase activity [27,28]. Enhanced calcium oscillations and altered expression of contractile proteins, such as myosin heavy chains, have also been reported [29]. Moreover, SMG stimulates nitric oxide (NO) production and upregulates inducible nitric oxide synthase (iNOS) gene expression in cardiomyocytes [30].

Despite these findings, there have been no straightforward investigations into ion currents through specific MGCs in cardiomyocytes under SMG conditions. Nonetheless, the decreased activity of mechanosensitive porins in *E. coli* [31], reduced membrane conductance in Xenopus oocytes [32,33], and membrane depolarization in neurons during microgravity [34] all provide background that suggests an influence of microgravity on the properties of mechanosensitive ion channels. SMG has particularly varying effects on MGCs in different tissues. For example, simulated microgravity (SMG) has been shown to upregulate L-type Ca^2+^ channel expression in cerebral vessels, while downregulating it in mesenteric vessels [35]. Additionally, SMG upregulates the expression of T-type Ca^2+^ channels in vascular smooth muscle cells (VSMCs) [36]. TRPC6 channel function was decreased in SMG-treated intervertebral disks [37], while Piezo1 expression shows divergent patterns across tissues, with decreased expression in bone marrow [38] and increased expression in endothelial cells [39]. Piezo1-mediated mechanotransduction has also been implicated in SMG-induced carotid artery remodeling [40] and skeletal muscle degradation [41]. Such tissue-specific effects are thought to relate to local fluid redistribution and the modification of mechanical load distribution by SMG, which selectively modulate the expression/function of channels between organs. Nevertheless, there is limited cardiomyocyte-specific data concerning the role of MGCs.

In this study, we attempted to address this gap by examining the molecular and electrophysiological consequences of prolonged SMG on rat ventricular cardiomyocytes. We performed RNA sequencing with TPM normalization to assess the expression profiles of genes encoding MGCs and MSCs, identifying significant alterations in transcript levels. For TRPM7, we verified diminished protein levels via Western blot. Finally, whole-cell patch-clamp measurements showed suppressed stretch-induced currents in single cardiomyocytes from SMG-exposed animals and indicated that SMG reduces cardiac mechanoelectrical feedback through disruption of MGC function.

## 2. Results

### 2.1. Simulated Microgravity Changes the Abundance of Protein Transcripts of MGCs, MSCs, and Nitric Oxide Synthase (NOS)

In ventricular cardiomyocytes from control rats not subjected to simulated microgravity (SMG), RNA sequencing revealed detectable transcriptional activity (expressed in TPM) in several non-selective cation mechanically gated channels (MGCs), including TMEM63A (0.231 ± 0.054), TMEM63B (2.533 ± 0.329), TRPC1 (0.205 ± 0.024), TRPV2 (0.068 ± 0.011), TRPM4 (0.270 ± 0.008), TRPM7 (0.236 ± 0.021), PKD1 (0.616 ± 0.111), PKD2 (0.300 ± 0.049), and Piezo1 (0.163 ± 0.048).

**Figure 1:**
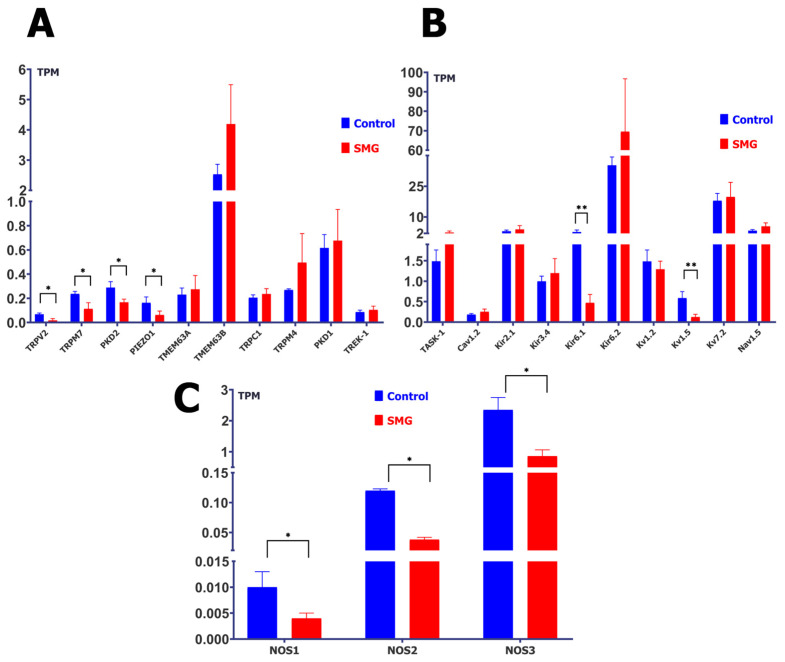
The effect of simulated microgravity (SMG) on transcript levels of mechanically gated channels (MGCs), mechanosensitive channels (MSCs), and nitric oxide synthases (NOS). Gene expression was quantified and normalized using the TPM (Transcripts Per Kilobase Million) method. (**A**): Expression of MGC-related transcripts under SMG. Blue bars represent control (*n* = 7; red bars represent SMG-treated samples (*n* = 4). * *p* < 0.05; all other comparisons not significant (NS). (**B**) Expression of MSC-related transcripts under SMG. Blue bars—control (*n* = 7); red bars—SMG (*n* = 4). ** *p* < 0.001; all other comparisons NS (Kv and Kir channels in this figure are presented under the category of MSCs due to their reported mechanosensitive modulation or physiological relevance to membrane responses under stretch, though they are not classical MGCs). (**C**) Expression of NOS isoforms under SMG. Blue bars—control (*n* = 7); red bars—SMG (*n* = 4). * *p* < 0.05.

Following 14 days of SMG exposure, the expression of several of these MGCs showed a statistically significant decrease (Figure 1A): TRPV2: 0.018 ± 0.015 (–74%, *p* < 0.05); TRPM7: 0.112 ± 0.029 (–52%, *p* < 0.05); PKD2: 0.167 ± 0.026 (–44%, *p* < 0.05); and Piezo1: 0.063 ± 0.032 (–61%, *p* < 0.05).

Conversely, other MGCs displayed a trend toward increased expression (Figure 1B): TMEM63A: 0.275 ± 0.114 (+19%); TMEM63B: 4.191 ± 1.303 (+65%); TRPC1: 0.236 ± 0.044 (+15%); TRPM4: 0.496 ± 0.246 (+84%); and PKD1: 0.676 ± 0.258 (+10%). While some changes were notable in magnitude, only those marked with asterisks in the figure reached statistical significance.

In a separate analysis, the expression of mechanosensitive potassium channels (MSCs) and potassium-selective MGCs was assessed (Figure 1C). Among them, Kir6.2 showed the highest transcript level in control cells (35.25 ± 4.07 TPM), which significantly increased after SMG exposure to 69.54 ± 27.18 TPM (+97%, *p* < 0.05). In contrast, Kir6.1 decreased markedly from 2.712 ± 0.943 to 0.472 ± 0.203 (–83%, *p* < 0.05).

Other representative channels and observed trends included Kv7.2: 17.976 ± 3.553 → 19.814 ± 7.130 (+10%); Nav1.5: 3.324 ± 0.508 → 5.447 ± 1.706 (+64%); Kir2.1: 3.048 ± 0.583 → 3.962 ± 1.837 (+30%); TASK-1: 1.485 ± 0.279 → 2.429 ± 0.761 (+64%); Kv1.2: 1.482 ± 0.281 → 1.292 ± 0.197 (–13%); Kir3.4: 0.997 ± 0.124 → 1.197 ± 0.354 (+20%); Kv1.5: 0.588 ± 0.158 → 0.126 ± 0.065 (–79%, *p* < 0.05); Cav1.2: 0.185 ± 0.024 → 0.253 ± 0.065 (+37%); and TREK-1: 0.087 ± 0.015 → 0.105 ± 0.030 (+21%).

It is important to note that not all changes reached statistical significance; these trends are presented to offer a comprehensive overview of potential transcriptional modulation under SMG conditions. The figure has been organized to reflect the classification of channels based on gating mechanism: MGCs are shown in panels A and B, while MSCs and potassium-selective channels are presented in panel C. This organization is intended to enhance clarity and facilitate integrative interpretation of the results.

Since MGCs function is under the control of NO, we measured the TPM levels for the intracellular sources of NOS. A trend towards decreased TPM was observed for all three known NOS isoforms (Figure 1C). NOS1 decreased by 60% (from 0.01 ± 0.003 to 0.004 ± 0.001), NOS2 decreased by 68% (from 0.12 ± 0.003 to 0.038 ± 0.004), and NOS3, which represented the largest amount of TPM, decreased by 63% (from 2.35 ± 0.400 to 0.860 ± 0.201).

### 2.2. SMG Causes a Decrease in TRPM7 Channel Protein

**Figure 2:**
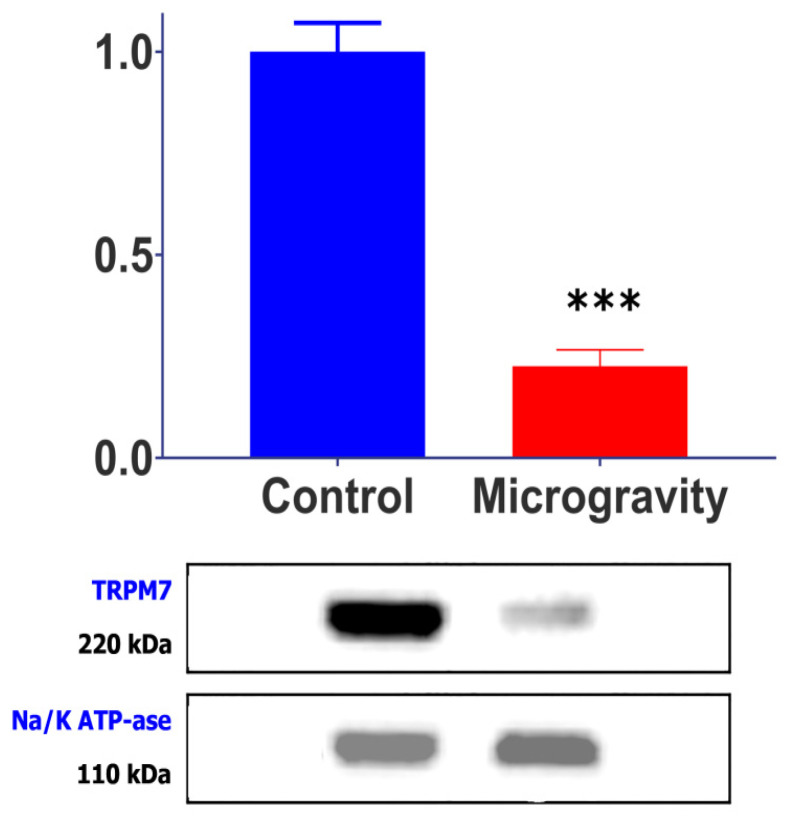
Cardiomyocyte TRPM7 channel protein levels normalized to housekeeping protein (α-subunit of Na^+^/K^+^-ATPase, validated as stable under SMG conditions by RNA-seq analysis, *p* = 0.89) in control animals (*n* = 11) and animals subjected to SMG (*n* = 8). *** *p* < 0.001 compared to control.

RNA-seq analysis confirmed stable expression of the Na/K-ATPase α-subunit under SMG conditions (TPM: control 45.2 ± 8.1 vs. SMG 43.8 ± 9.4; *p* = 0.89), validating its use as a housekeeping control for Western blot analysis. Western blot analysis, conducted to test the correspondence between changes in the TPM of channels during prolonged microgravity and the corresponding change in the amount of channel protein, showed that the content of the MGCs TRPM7 protein in cardiomyocytes was significantly lower in rats from the SMG group than those in the control group (Figure 2). When the amount of this protein in cardiomyocytes from the control group of rats was normalized to housekeeping, protein was taken as 1 (1 ± 0.251; *n* = 11); in cardiomyocytes from the experimental group it was recorded as 0.233 ± 0.095 relative units (*n* = 8; *p* < 0.001). Correspondingly, TPM for TRPM7 also decreased, from 0.236 ± 0.021 in the control to 0.112 ± 0.029 (by 52%), in cardiomyocytes of rats from the SMG group. The α-subunit protein of Na/K-ATPase, whose amount did not change under the influence of SMG, was used as the housekeeping protein.

### 2.3. SMG Alters I_MGC_ Sensitivity to Stretch

We recorded the late membrane current (I_L_), defined as the net current measured at the end of each 140 ms voltage pulse. This current reflects the superimposed contributions of three main components: (1) I_ns_, the current mediated by stretch-activated nonselective cation channels; (2) I_K1_, the inwardly rectifying potassium current; and (3) I_out_, which represents the sum of various outwardly rectifying currents. These may include potassium currents through two-pore domain (K_2P_, e.g., TREK) potassium channels [42] or other outwardly rectifying channels [43,44].

To further quantify the mechanically evoked response, we introduced the mechanically induced current (I_MIC_), defined as the difference between the late current recorded under stretch conditions (^S^I_L_) and that recorded in the absence of stretch (^C^I_L_). This differential current, I_MIC_ = ^S^I_L_ − ^C^I_L_, reflects the composite activity of mechanosensitive conductances, specifically the current through MGCs and MSCs. Differential current values are determined as the difference between control current values (^C^I_L_) and current values under conditions of cell stretch (^S^I_L_), specifically at −80 mV, and are designated as ΔI_L_ or I_MGC_. A differential current resulting from a shift in I_L_ values in a more negative direction relative to the reference is indicated by a minus (−), whereas a shift in a more positive direction is indicated by a plus (+).

**Figure 3:**
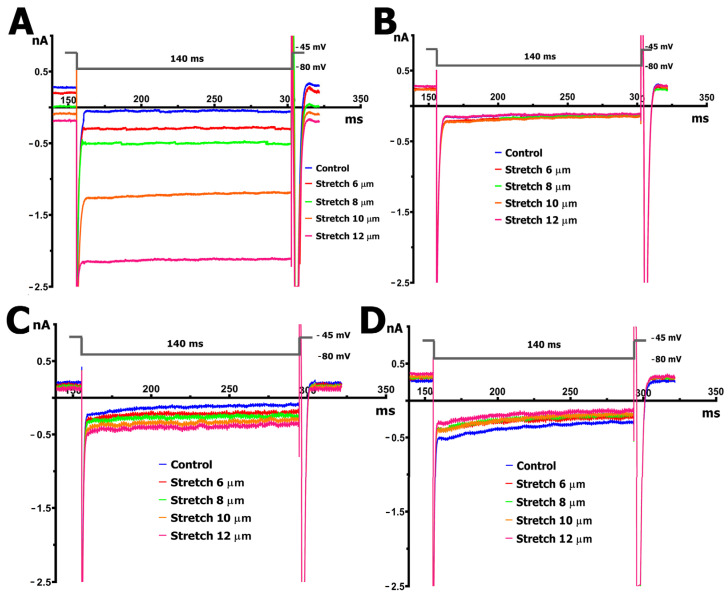
Original recordings of the membrane currents at a holding potential of −45 mV with a 140 ms step to −80 mV before cell stretching (blue curve) and upon stretching by 6 (red curve), 8 (green curve), 10 (orange curve), and 12 (pink curve) µm. (**A**) Shift in the currents towards negative values upon the graded stretching of cardiomyocytes from control rats. (**B**) No change in the currents upon the graded stretching of cardiomyocytes from rats exposed to SMG. (**C**) Decreased shift in the currents towards negative values (compared to control rats in “A”) upon the graded stretching of cardiomyocytes from rats exposed to SMG. (**D**) Slight shift in the currents towards positive values upon the graded stretching of cardiomyocytes from rats exposed to SMG.

Representative original traces from whole-cell patch-clamp recordings in cardiomyocytes under control and SMG conditions, before and during mechanical stretch, are shown in Figure 3. These traces illustrate the heterogeneity of I_MGC_ responses. The currents reflect a composite profile involving multiple MGCs, not limited to TRPM7.

**Figure 4:**
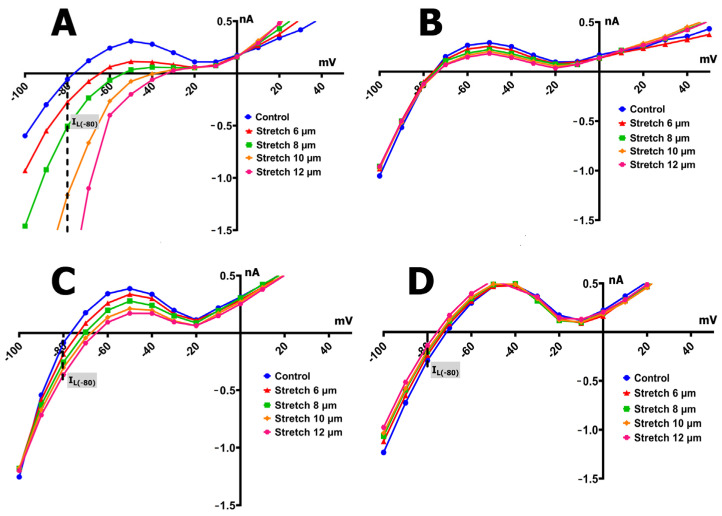
Voltage dependence of the I_L_ recorded in ventricular cardiomyocytes from control rats and rats exposed to SMG, before and during graded mechanical stretching. I_L_ reflects the net I_MIC_, comprising contributions from I_MGC_ and I_MSC_. Blue curves indicate control (unstretched) conditions; red, green, orange, and pink curves represent stretches of 6, 8, 10, and 12 µm, respectively. (**A**) Control group: I_L_ increases progressively with stretch, especially at negative potentials, indicating the robust activation of mechanosensitive currents. (**B**) SMG group—type I response: no significant change in I_L_ across stretch levels, indicating loss of stretch sensitivity. (**C**) SMG group—type II response: modest increase in I_L_ with stretch, but significantly attenuated compared to control. (**D**) SMG group—type III response: I_L_ decreases with stretch, suggesting a paradoxical or reversed response to mechanical loading.

**Figure 5:**
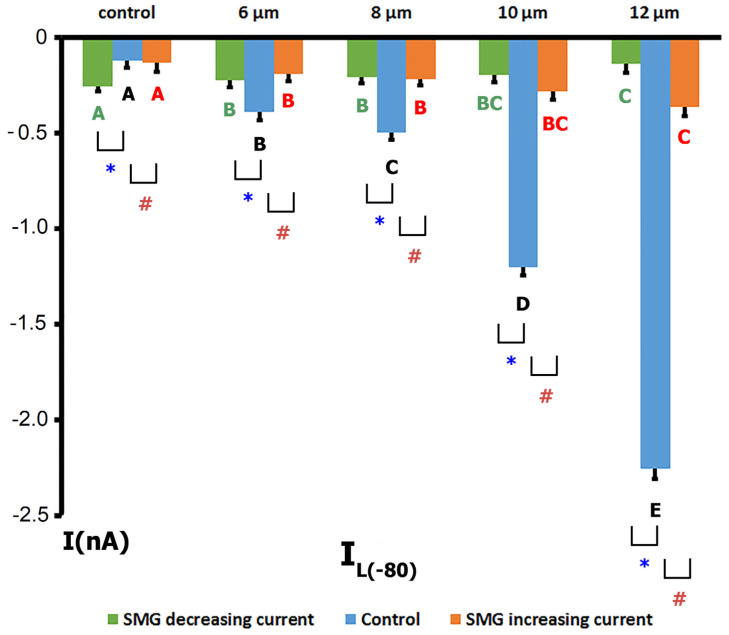
Bar graph showing mean values of I_L_ at −80 mV in ventricular cardiomyocytes from control rats (blue bars; *n* = 9) and rats exposed to SMG. I_L_ values were measured under baseline conditions and during graded mechanical stretching (6, 8, 10, and 12 µm). The SMG group is subdivided into two response phenotypes: cells with a slight increase in I_L_ (orange bars; *n* = 8, corresponding to Figure 3C) and those with a decrease in I_L_ (green bars; *n* = 6, corresponding to Figure 3D). Letters (A–E) above bars of the same color indicate statistically significant differences between stretch levels within a group (*p* < 0.01); identical letters denote no significant difference (NS). Asterisks (*) and hash marks (#) indicate statistically significant differences between control and SMG groups at the corresponding stretch level (*p* < 0.01).

**Figure 6:**
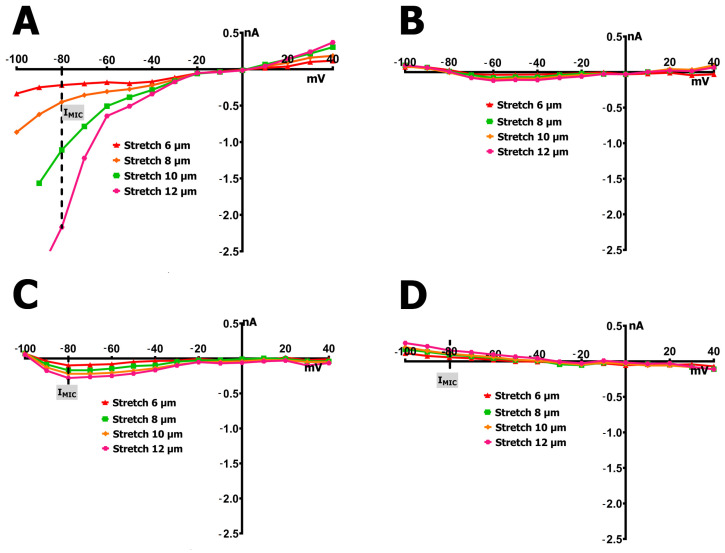
Voltage dependence of the net mechanically induced current (I_MIC_) in ventricular cardiomyocytes from control rats and those exposed to SMG, calculated as the difference between late currents recorded under stretch (^S^I_L_) and control conditions (^C^I_L_): I_MIC_ = ^S^I_L_ − ^C^I_L_. Each panel shows I_MIC_ at −80 mV across increasing stretch amplitudes: 6 µm (red), 8 µm (green), 10 µm (orange), and 12 µm (pink). (**A**) Control group: robust, progressive increase in inward I_MIC_ with stretch, indicating normal mechanosensitivity. (**B**) SMG group—type I response: complete absence of stretch-induced current, reflecting loss of mechanotransductive response. (**C**) SMG group—type II response: attenuated I_MIC_ compared to controls, suggesting reduced mechanosensitivity. (**D**) SMG group—type III response: paradoxical positive I_MIC_ values, indicating reversed response to mechanical stretching.

The *I/V* curves in Figure 4A and the corresponding blue bars in Figure 5 illustrate the voltage dependence of the I_L_ in control cardiomyocytes, along with its modulation through mechanical stretching. The I_MIC_ responses at various stretch amplitudes in the control group are shown in Figure 6A.

Under baseline (unstretched) conditions, the *I/V* relationship of the I_L_ exhibited an N-shaped profile and intersected the voltage axis (zero current potential, V_0_) at approximately −78 mV (−76  ±  3 mV, *n* = 9), consistent with the estimated resting potential of non-clamped cells (Figure 4A). The control current at −80 mV (^C^I_L_) was −0.09  ±  0.04 nA (Figure 5).

The application of a modest 6 µm stretch depolarized V_0_ to −66  ±  3 mV and shifted I_L_ toward more negative values, with ^S6^I_L_ reaching −0.30  ±  0.045 nA. This yielded a differential current (I_MIC_) of −0.26  ±  0.06 nA (^S6^I_MGC_, *n* = 9). The negative sign indicates that stretch caused an increase in the inward (negative) current, shifting the *I/V* curve downward. Notably, the pre- and post-stretch *I/V* curves crossed near 0 mV, and I_L_ values were increased at positive voltages, reflecting enhanced outward currents.

Further increases in stretch produced graded changes in V_0_ and I_L_:

- An 8 µm stretch depolarized V_0_ to −55  ±  3 mV and increased I_L_ to −0.498  ±  0.036 nA, corresponding to a differential current of −0.411  ±  0.050 nA (*n* = 9; ^S8^I_MGC_).
- A 10 µm stretch shifted V_0_ to −41  ±  3 mV and ^S^I_L_ to −1.205  ±  0.042 nA, with a resulting ^S10^I_MGC_ of −1.12  ±  0.063 nA (*n* = 8).
- A 12 µm stretch caused the most pronounced depolarization, with V_0_ reaching −31 ± 3 mV and ^S^I_L_ increasing to −2.256 ± 0.053 nA. The corresponding differential current was −2.23 ± 0.055 nA (*n* = 6; ^S12^I_MGC_).

These results collectively demonstrate that, in control cells, graded mechanical stretch induces a voltage-dependent increase in I_L_, predominantly through the activation of mechanically gated channels.

Cardiomyocytes from rat ventricles exposed to SMG demonstrated three types of response to stretch: in the first case, there was no response to stretch at all, i.e., ^S^I_L_ did not change compared to ^C^I_L_ (Figure 4B), so there was no I_MIC_ in this case (Figure 6B). In the second case, a slight increase in ^S^I_L_ was observed (Figure 4C and Figure 5 orange columns) as well as modest negative I_MIC_ (Figure 6C), and in the third case, a slight decrease in ^S^I_L_ was observed compared to the control (Figure 4D and Figure 5 green columns) with a modest positive I_MIC_ (Figure 6D).

In more detail, in 30% (*n* = 6; *m* = 3) of cases, upon the discrete stretching of isolated cardiomyocytes from SMG rats by 6, 8, 10, and 12 µm, no changes in I_L_ at the level of −80 mV occurred, as noted above (Figure 4B and Figure 6B). The *I/V* curve of ^C^I_L_ intersected the potential axis at −77 mV (−77 ± 2 mV, *n* = 6). The ^C^I_L_ value at −80 mV in the control was −0.132 nA (−0.126 ± 0.053 nA), which was greater than that of cardiomyocytes from control rats. However, ^S6^I_L_, ^S8^I_L_, ^S10^I_L_, and ^S12^I_L_ did not change statistically significantly and were −0.102 nA (−0.105 ± 0.040 nA), −0.124 nA (−0.118 ± 0.051 nA), −0.139 nA (−0.137 ± 0.032 nA), and −0.124 nA (−0.125 ± 0.041 nA), respectively. It should be noted that a small discrete decrease in I_K1_ was recorded in response to stretching by 6–12 µm.

In 40% (*n* = 8; *m* = 3) of the total number of experiments, with the discrete stretching of cardiomyocytes of rats from the SMG group by 6–12 µm, an increase in I_L_ was recorded, but its values were significantly smaller (Figure 4C and Figure 5 orange columns) than those of the control group (Figure 4A and Figure 5 blue columns); as a result, the values of I_MIC_ were also significantly smaller than those of the control group (Figure 6C).

In cardiomyocytes of rats from the SMG group, before stretching, the *I/V* curve of ^C^I_L_ crossed the potential axis (zero current potential V_0_) at −78 mV (−76 ± 2 mV). Figure 4C shows that the ^C^I_L_ value at −80 mV in the control was −0.091 nA (−0.13 ± 0.05 nA). Stretching the cell by 6 µm shifted V_0_ toward depolarization to −76 mV (−75 ± 3 mV) and shifted ^C^I_L_ to more negative values. ^S6^I_L_ became −0.184 nA (−0.190 ± 0.041 nA). Thus, the differential current of ^S6^I_MGC_ was (−)0.093 nA ((−)0.103 ± 0.05 nA). Stretching by 8 µm shifted V_0_ to −73 mV (−72 ± 2 mV) and changed the current slightly, shifting ^C^I_L_ to ^S8^I_L_, which was equal to −0.254 nA (−0.220 ± 0.031 nA). ^S8^I_L_ and ^S6^I_L_ were not statistically significantly different. Stretching by 10 µm shifted V_0_ to −68 mV (−69 ± 3 mV) and shifted ^C^I_L_ to ^S10^I_L_, which was equal to −0.304 nA (−0.284 ± 0.04 nA). ^S6^I_L_, ^S8^I_L_, and ^S10^I_L_ were not statistically different. Stretching by 12 μm shifted V_0_ to −65 mV (−65 ± 2 mV) and shifted ^C^I_L_ to ^S12^I_L_, equal to −0.364 nA (−0.366 ± 0.047 nA). Thus, the differential current ^S12^I_MGC_ became equal to (−)0.279 nA ((−)0.273 ± 0.055 nA). ^S12^I_L_ did not differ significantly from ^S10^I_L_, but differed from ^S8^I_L_ and ^S6^I_L_. Therefore, in this case, the current for stretching the cell by 12 μm (^S12^I_MGC_) was approximately equal to the current for stretching by 6 μm, as recorded in the control series of experiments (^S6^I_MGC_).

In another 30% (*n* = 6; *m* = 3) of cases, in cardiomyocytes of rats from the SMG group, a slight decrease in ^S^I_L_ was observed in comparison with the control current I_L_ when gradually stretched by 6–12 µm. The *I/V* curves in Figure 4D and the green columns in Figure 5 show the changes in I_L_ in this group of animals; Figure 6D represents the changes in I_MIC_. In the cardiomyocytes of rats from the SMG group before stretching, the *I/V*-curve ^C^I_L_ crossed the potential axis (zero current potential V_0_) at −73 mV (−75 ± 2 mV). The ^C^I_L_ value at −80 mV in the control was equal to −0.291 nA (−0.255 ± 0.04 nA), i.e., significantly higher in absolute value than in the control group. Stretching the cell by 6 μm shifted V_0_ towards hyperpolarization to −74 mV (−75 ± 2 mV) and shifted ^C^I_L_ to more positive values; ^S6^I_L_ became equal to −0.234 nA (0.222 ± 0.047 nA). Thus, the differential current of ^S6^I_MGC_ was equal to (+)0.057 nA ((+)0.07 ± 0.021 nA). Stretching by 8 μm shifted V_0_ to −75 mV (−76 ± 2 mV) and shifted ^S6^I_L_ to ^S8^I_L_, which became equal to −0.197 nA (−0.210 ± 0.029 nA); ^S6^I_L_ and ^S8^I_L_ showed no significant statistical differences. A 10 μm stretch shifted V_0_ to −76 mV (−77 ± 3 mV) and shifted ^S8^I_L_ to ^S10^I_L_ of −0.184 nA (−0.195 ± 0.04 nA). ^S6^I_L_, ^S8^I_L_, and ^S10^I_L_ were not statistically different. A 12 μm stretch shifted V_0_ to −77 mV (−77 ± 2 mV) and shifted ^S10^I_L_ to ^S12^I_L_ of −0.137 nA (−0.140 ± 0.043 nA). ^S12^I_L_ was not significantly different from ^S10^I_L_, but was different from ^S8^I_L_ and ^S6^I_L_. Thus, the differential current ^S12^I_MGC_ became equal to (+)0.154 nA ((+)0.145 ± 0.051 nA); that is, ^S12^I_L_ became almost equal to ^C^I_L_ in the control group.

**Figure 7:**
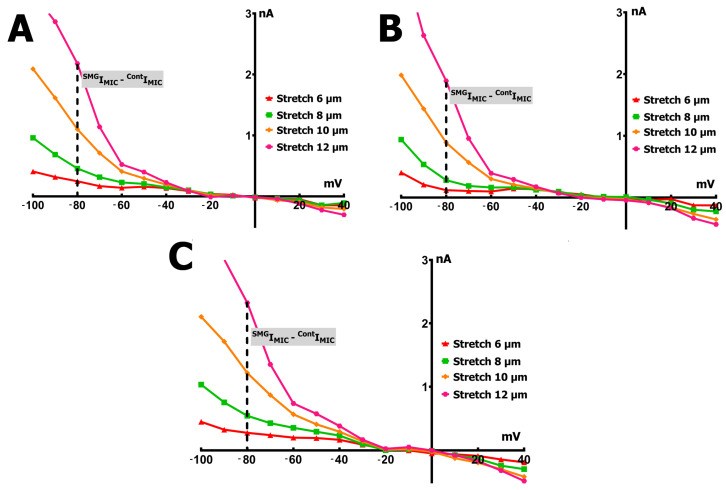
Difference in net I_MIC_ between cardiomyocytes from SMG rats and control rats, plotted as ^S^I_MIC_ − ^C^I_MIC_ across membrane potentials. Curves represent stretch amplitudes of 6 µm (red), 8 µm (green), 10 µm (orange), and 12 µm (pink). (**A**) Type I response: no significant change in I_MIC_ across stretch levels, indicating preserved absence of mechanosensitive current in SMG cardiomyocytes. (**B**) Type II response: consistently reduced I_MIC_ in SMG cells compared to controls, reflecting diminished stretch sensitivity. (**C**) Type III response: positive shift in I_MIC_ difference, consistent with reversed (paradoxical) response to stretch under SMG conditions.

Figure 7 illustrates the difference in mechanically induced current (I_MIC_) between cardiomyocytes from SMG-exposed rats and controls (^SMG^I_MIC_ − ^C^I_MIC_) following graded stretching at 6, 8, 10, and 12 µm. Across all response types, I_MIC_ was consistently lower in the SMG group, with the most pronounced differences observed at greater stretch amplitudes. These findings highlight a marked attenuation of stretch sensitivity in cardiomyocytes under SMG conditions.

## 3. Discussion

The main change that occurs in the body under the influence of MG in space is the redistribution of body fluids. In the absence of gravity, venous return to the heart increases, which in turn leads to an increase in the end-diastolic volume; consequently, cardiomyocytes are in a state of greater stretch than under normal conditions [16].

To simulate the processes occurring in the body under weightlessness (under spaceflight conditions), methods are used that include horizontal bed rest, bed rest with head-down tilt, immersion in water with head exposed, and dry immersion with head off (immersion with an impermeable barrier of elastic fabric between the subject and water) [45].

In animal experiments, to study the molecular and cellular mechanisms of the cardiovascular system, the simulation of microgravity in the form of unloading the hind limbs of rodents is mainly used [46].

Physiological changes that occur in the body will be either a direct consequence of the effect of SMG or compensatory mechanisms designed to counteract such influence. Primarily, these changes are expected to influence molecular processes that play a role, to some degree, in the body’s response to SMG. In relation to the heart, these changes mainly involve alterations in the transcription of genes encoding MGCs, MSCs, and other ion channels, variations in the number of channel proteins, and consequently, changes in ion currents.

### 3.1. Simulated Microgravity Affects the Gene Expression of Cardiomyocyte MGCs and MSCs Proteins

Previously, all channels identified as MGCs and MSCs were described in [12]; in this work, we focused on these data.

For several types of MGCs, including TRPM7, TRPV2, PKD2, Piezo1, a decrease in the number of transcripts of their genes is observed. Of these channels, TRPM7 stands out as the most represented in terms of the TPM level in the control and in terms of the dynamics of the decrease in its transcripts under the influence of SMG. TRPM7 is a full-fledged ion channel for divalent cations at negative potentials and allows the current of monovalent ions only during depolarization [47]. The channel selectivity profile is Zn = Ni > Ba > Co > Mg > Mn > Sr > Cd > Ca [48,49]. Under physiological conditions, TRPM7 channels control permeability for Ca^2+^ and Mg^2+^ [50]. The TRPM7 channel is directly activated by cell stretching or other mechanical action [51]. Interestingly, in patients with ischemic cardiomyopathy, a direct relationship was shown between decreased TRPM7 expression in the left ventricle and decreased ejection fraction [52], i.e., a similar dysfunction was observed to that often observed with MG exposure. A correlation was also found between decreased TRPM7 expression and increased cytoplasmic Ca^2+^ concentration in H9C2 cells during hypoxia [53].

The results of our experiments clearly demonstrate a simultaneous decrease in both TPM for TRPM7 (from 0.236 ± 0.021 in the control to 0.112 ± 0.029 (by 52%)) after SMG, and its channel protein. The conducted Western blot showed a reliable and significant decrease in the protein content of this channel to 0.23 ± 0.09 relative units (*n* = 8; *p* < 0.001) in cardiomyocytes after exposure to SMG. These data are in good agreement with our electrophysiological results, which showed a decrease in I_MGC_ sensitivity to stretch. Thus, these studies confirm our assumption about the molecular mechanism of this phenomenon: as a result of SMG, changes in the number of transcripts of MGC genes lead to co-directional changes in the synthesis of channel proteins, which directly affects the final ionic conductivity of these channels.

Although TRPM7 is not a classical stretch-activated channel in the strict biophysical sense, previous studies and our current findings support its functional involvement in mechanotransduction pathways under specific pathophysiological conditions such as hypoxia and SMG [48,49,50,51,52,53]. Importantly, the current observed in our patch-clamp experiments—termed I_MGC_—should not be attributed solely to TRPM7. The observed current profile is likely generated by a composite activity of multiple non-selective cationic MGCs, including Piezo1, PKD2, and TRPV2, whose transcript levels were also altered under SMG.

Another channel of interest for our study is the Piezo1 channel, which plays a key role in the mechanosensitivity of skeletal muscle and is involved in changing its function under the influence of MG [41]. We found a reliable decrease in the expression of Piezo1 in the cardiomyocytes of rat ventricles. Interestingly, it was recently shown that Piezo1 in the mouse heart is a mechanosensor responsible for the development of cardiac hypertrophy under pressure-overload conditions [54]. Since a similar effect is observed under MG and SMG conditions due to the redistribution of fluid in the body, it can be assumed that a decrease in the expression level of this channel is a compensatory reaction of the body aimed at preventing cardiac hypertrophy.

We also showed a decrease in TPM for the PKD2 (TRPP2) protein [55]. Interestingly, PKD2 is able to form a functional cation channel with PKD1 and TRPC1 [55], which complicates the interpretation of the increase in expression of these proteins shown in this study.

Finally, we observed a decrease in the TPM level for the TRPV2 channel. It is important to note that in this case the connection between the decrease in TPM and the number of functional channels is even less obvious than it normally is, since, in most cells, these channels are located on the ER and are translocated to the plasma membrane only upon the stimulation of PI3K and mechanical action [52]. In cardiomyocytes, the role of TRPV2 in pathophysiology was demonstrated—in cells from animals with muscular dystrophy, the overexpression of TRPV2 was shown, which led to an increase in the plasma concentration of Ca^2+^. A decrease in the number of functional TRPV2 on the membrane led to a decrease in the severity of dystrophic signs [55]. Perhaps, a similar compensatory role is played by the decrease in the expression of channels of this type under the influence of SMG.

For transcripts of some other MGCs, such as TMEM63A, TMEM63B, TRPC1, TRPM4, and PKD1 (TRPP1), we showed a tendency for TPM to increase after exposure to SMG, which at first glance may be at odds with the electrophysiological data. However, it is worth noting that the synthesis of the channel protein itself does not necessarily mean the formation of a functional ion channel. This is precisely the situation that is possible with the TMEM63B protein, whose transcripts are present in large quantities in the control and are increased in the MG group. In a study by Zhao and colleagues, it was shown [56] that only when all three types of murine TMEM63 channel proteins were co-expressed in HEK 293 cells did an osmotically induced ion current appear, and the absence of at least one TMEM63 subtype completely eliminated this current. In our case, the content of TMEM63A transcripts was an order of magnitude lower than TMEM63B, and TMEM63C was negligible, so it is likely that the presence of a large number of transcripts of this protein type does not mean that a large number of ion channels are formed by them.

We also showed a tendency toward an increase in the number of PKD1 (TRPP1) channel transcripts. Interestingly, one of the functions of this channel protein in cardiomyocytes is assumed to be decreasing the probability of ryanodine receptor 2 (RYR2) opening [57], i.e., decreasing the release of Ca^2+^ ions from the depot, which may be part of a safety mechanism when the load on the heart increases during MG due to decreased cardiomyocyte contractility.

We also observed a tendency for an increase in the TPM of the TRPM4 channel protein. An increase in the mRNA content of this channel was observed by other authors during 4 weeks of intensive training in mice on a treadmill [58]—another situation where the heart experiences increased preload due to increased venous return. The authors suggest a protective function of TRPM4 against the pathological remodeling of the heart under loads associated with the phosphatidyl–inositol 3-kinase/Akt, (PI3K/Akt) pathway. The activation of these channels also leads to a decrease in the entry of Ca^2+^ into the cell, as in the case of PKD1 (TRPP1), but through store-operated channels.

In humans, channels with a two-pore domain TASK-1 in the heart are mainly expressed in atrial cardiomyocytes, where they participate in shortening the action potential (AP) and, therefore, perform an antiarrhythmogenic function [59]. However, in rats, these channels are also present in the ventricles, which was demonstrated in this study and by other authors [60]. It can be assumed that the tendency we recorded to increase the expression of these channels under the action of SMG serves as a protective mechanism against chronically prolonged AP in cells subject to greater than normal stretching [58], preventing extrasystole. It was also shown that an increase in TASK-1 expression leads to increased development of hypertrophy and cardiac dysfunction [61].

MSCs Kir6.1 and Kir6.2 are pore-forming subunits of ATP-sensitive potassium channels (K_ATP_)—the linking elements between the electrical properties of the cell and its metabolism, and the most important protectors in ischemia [62]. RNA sequencing showed that TPM for Kir6.2 was the most represented among all MGCs and MSCs (35.25 ± 4.066), the number of which significantly increased, by 97% (69.54 ± 27.18), under the influence of SMG. Such an increase can be considered as a protective mechanism under the increasing heart load in SMG conditions (see above). Kir6.1 was initially represented by a significantly smaller number of TPMs than Kir6.2, and SMG further reduced their number by 83%.

Voltage-gated channels (VGCs) K_v_7 represent potassium-selective voltage-gated channels (K^+^-VGCs), which in the heart are responsible for the slow component of the delayed rectifier current I_Ks_ [63]. This study demonstrated that the TPM of the K_v_7.2 channel was present in substantial quantities (17.976 ± 3.553), and SMG resulted in a 10% increase (19.814 ± 7.130). This observation likely indicates a cardioprotective effect, as the elevation in channel TPM can lead to increased protein abundance, consequently enhancing the repolarizing current and shortening the action potential duration (APD).

K_ir_2.1 channels belong to the canonical inward-rectifier potassium (K^+^) channels, which are responsible for the I_K1_ current and, consequently, for maintaining the resting membrane potential of cardiomyocytes [64]. SMG increased the TPM of K_ir_2.1 by 30% (from 3.048 ± 0.583 to 3.962 ± 1.837), which potentially could lead to an increase in the number of these channels and stabilization of the resting potential at a lower level, and, consequently, contribute to decreased cardiomyocyte excitability.

K_ir_3.4 channels also belong to the inward-rectifier potassium channels and, in cardiomyocytes, are responsible for the I_K,Ach_ current, thereby mediating an increase in potassium conductance under the influence of the parasympathetic nervous system [64]. The TPM levels of these channels also experienced a modest rise due to the influence of SMG.

TPM of the TREK-1 channel was present in small quantities; however, it increased by 21% under the influence of SMG. TREK-1 is a two-pore domain K^+^-channel, which has been shown to be the main potassium MGC in cardiomyocytes [65]. The elevated gene expression of this channel induced by SMG may also serve a protective function by enhancing potassium conductance in cardiomyocytes during conditions of increased preload associated with SMG.

The only potassium channel whose TPM was shown to decrease under the influence of SMG was the voltage-gated K_v_1.5. It is known that this channel is of great importance for atrial cardiomyocytes and is responsible for the formation of the ultrafast rectifier current I_Kir_ there; however, in ventricular cardiomyocytes, the amount of protein in this channel is extremely small, and this current has not been shown [66,67].

Although Kir and Kv channels are not classified as canonical mechanically gated channels, some subtypes (e.g., Kir2.1, Kv1.2) have been reported to exhibit mechanosensitive modulation under specific conditions [64]. Their inclusion in our dataset is based on their altered expression under SMG and their key roles in maintaining membrane excitability, rather than on direct mechanogating mechanisms.

Ca_v_1.2 is responsible for the L-type Ca^2+^ current and, accordingly, for the entry of Ca^2+^ into the cardiomyocyte during the plateau phase of the AP [68]. SMG increases the TPM of these channels by 37%, which may play a role in the increase in stroke volume under SMG, as has been shown in some studies [23].

Nav1.5 is an isoform of the VGCs Na^+^ channel expressed in ventricular cardiomyocytes and responsible for the depolarization phase of the AP [69]. We have shown that the TPM of these channels increases by 64% under the influence of SMG.

Thus, such a multidirectional change in the TPM of proteins of different MGCs and MSCs can lead to the activation of a particular adaptive process associated with various molecular pathways that include a certain channel.

### 3.2. SMG Changes the Electrical Response to Stretch

Under control conditions, it was shown that local axial stretching of cardiomyocytes causes changes in the electrical properties of the cell membrane, which are most pronounced in late I_L_ currents. The change in current during the stretching of cardiomyocytes is expressed in the appearance of a differential current I_MGC_. As shown in previous studies, I_MGC_ is not a Ca^2+^-activated current (no effect of preloading of the cells with BAPTA) and that Cl^−^ fluxes do not contribute to I_MGC_ (no effect of changing Cl^−^ gradients). Together with the known properties, such as the linear voltage dependence, the E_rev_ of 0 mV, and the sensitivity to Gd^3+^, our results indicate that I_MGC_ flows through non-selective cation MGCs [6].

It has also been shown that I_MGC_ is the basis for stretch-related membrane potential depolarization (mechanically induced depolarization), which is known to lead to a change in the resting potential and prolongation of the action potential and can cause afterdepolarization and extra APs [6].

Cardiomyocytes from rat ventricles exposed to SMG exhibited three distinct types of electrophysiological responses to stretch. In the first, no change was observed in the ^S^I_L_ compared to the ^C^I_L_, indicating complete loss of stretch sensitivity. The second group showed a modest increase in ^S^I_L_, while the third displayed a paradoxical reduction in ^S^I_L_ relative to the control.

These divergent responses may result from multiple overlapping factors One possible explanation involves variation in the expression of mechanosensitive ion channel (MGC) proteins, including TRPM7, whose abundance was found to be altered—either directly observed or inferred—in our study. Another potential factor involves post-translational modifications of channel proteins, influenced by changes in second messenger systems under SMG. We previously demonstrated that SMG significantly alters the expression of genes encoding soluble guanylate cyclases (sGCs), adenylate cyclase isoforms, and several phosphodiesterases (PDEs) in ventricular cardiomyocytes [70,71]. These changes may in turn affect channel phosphorylation states.

Additionally, SMG has been shown to modulate NO signaling, a key regulator of MGC function. Although NOS1 and NOS2 transcripts exhibited low TPM values, these isoforms are known to exert potent functional effects even at low expression levels due to their strategic subcellular localization and inducibility under stress conditions [72,73]. Previous studies, including our own, have reported that SMG reduces intracellular NO levels in cardiomyocytes [30] and leads to decreased transcript levels of all three NO synthase (NOS) isoforms, including an ~63% reduction in NOS3 transcript levels (TPM) [74,75]. Other work has confirmed decreased nNOS expression and NO production in skeletal muscle under SMG [75]. Because NO plays a dual role—facilitating MGC-opening when closed and promoting closure when MGCs are already open [76]—a reduction in NO could disrupt the dynamic regulation of these channels.

These findings are supported by earlier work showing that NO donors such as S-nitroso-N-acetylpenicillamine (SNAP) and Diethylamine NONOate (DEA-NO) enhance MGC and MSC currents in cardiomyocytes [74,76], whereas inhibition of NO production (via NOS blockers like ^N^G-Nitro-L-arginine methyl ester (L-NAME) or scavengers like 2-Phenyl-4,4,5,5-tetramethylimidazoline-1-oxyl 3-oxide (PTIO) abolishes the stretch response [77]. Similar effects have been observed in NOS3^−^/^−^ transgenic mice [77]. Thus, the reduced stretch sensitivity under SMG may result not only from downregulated MGC expression but also from an NO-deficient intracellular environment. Therapeutically, enhancing NOS activity or using NO donors may help restore stretch responsiveness in SMG-exposed cells.

The existence of three I_L_ response types may reflect the variable contributions of these molecular mechanisms. For instance, in cells where I_L_ decreased upon stretch (contrary to the normal increase), baseline I_L_ was significantly elevated, possibly indicating a larger pool of constitutively open MGCs. In these cells, stretch-induced NO production may trigger channel closure, in line with our previous findings that NO exerts opposing effects depending on the channel’s pre-activation state [76].

From a physiological perspective, these heterogeneous responses may represent different adaptive strategies to the altered mechanical environment of SMG. A lack of response may indicate impaired preload sensing and could contribute to a functional decline or cardiac atrophy. A modest increase in stretch-induced current may signify partial retention of mechanotransducive signaling, while a paradoxical decrease may reflect pathological remodeling or dysregulation of inhibitory pathways, such as excessive NO signaling.

Collectively, these results suggest that SMG induces differential molecular and functional remodeling in cardiomyocytes, leading to heterogeneous mechanoelectrical behavior. This variability may contribute to disrupted cardiac conduction or contractility in vivo and underscores the complexity of the heart’s adaptive responses to mechanical unloading. Rather than uniformly suppressing MGC function, SMG appears to generate a spectrum of functional states with both physiological and potentially pathological implications.

## 4. Materials and Methods

### 4.1. Animals

The experiments were carried out following the Guide for the Care and Use of Laboratory Animals (8th edition, 2011) issued by the US National Institutes of Health. The experimental protocol received approval from the Ethics Committee of the Russian National Research Medical University (protocol code # 27/2023 issued 25 December 2023). Male outbred Wistar rats, 8 weeks old and weighing between 200 and 220 g, were utilized for the experiments. The rats were kept on a 12:12 h light/dark cycle and had unrestricted access to food and water.

### 4.2. Simulation of Microgravity

In a microgravity simulation with controlled conditions, a widely used model for studying the cardiovascular system involves the unloading of rodents’ hind limbs [46]. In this setup, the rodent is suspended by its tail from the ceiling of its cage, allowing its forelimbs to rest on the floor while its hind limbs are elevated at an angle of 30° to 40° from the ground, without making contact with it. The animal retains the ability to move freely within the cage. In our experiments, rats were exposed to simulated microgravity for 14 days. Notably, under these circumstances, the corticosterone levels, which serve as a measure of the animal’s stress response, did not surpass those seen in control animals.

### 4.3. Solutions

Ca^2+^-free physiological salt solution (Ca^2+^-free PSS) was used, containing (in mmol/L): 118 NaCl, 4 KCl, 1 MgCl_2_, 1.6 NaH_2_PO_4_, 24 NaHCO_3_, 5 sodium pyruvate, 20 taurine, and 10 glucose; the solution was adjusted to pH 7.4 with NaOH and bubbled with carbogen (95% O_2_ + 5% CO_2_) [6,46]. Enzyme medium contained Ca^2+^-free PSS supplemented with 10 μmol/L CaCl_2_, 0.2 mg/mL collagenase (Type II, Worthington, 225 units/mg), and 1 mg/mL bovine serum albumin (Sigma-Aldrich, St. Louis, MO, USA) [6,78]. Prior to the actual experiments, cells were kept in a modified Kraftbrühe (KB) medium for a minimum of 2 h, which contained(mmol/L) 50 L glutamic acid, 30 KCl, 3 MgSO_4_ × 7H_2_O, 20 taurine, 10 glucose, 30 KH_2_PO_4_, 0.5 EGTA, and 20 HEPES, adjusted to pH 7.3 with KOH [6]. The isolated cells were preserved in a KB medium for as long as 8 h. Ventricular cardiomyocytes were perfused with a solution at 37 °C that contained the following (mmol/L): 150 NaCl, 5.4 KCl, 1.8 CaCl_2_, 1.2 MgCl_2_, 20 glucose, and 5 HEPES, at pH 7.4, adjusted with NaOH (K^+^_out_ solution). Internal pipette solution contained (mmol/L): 140 KCl, 5 Na_2_ATP, 5 MgCl_2_, 0.01 EGTA, 10 Hepes/KOH at pH 7.3 (K^+^_in_ solution). Further in the text, this configuration is called K^+^_in_/K^+^_out_ solution.

### 4.4. Isolated Cardiomyocyte Preparation

We utilized a modified version of the previously described cell isolation procedure [6]. Ventricular myocytes were dispersed using the standard collagenase dissociation technique. Briefly, the isolated rat hearts were perfused retrogradely (37 °C), allowing for a constant flow of 1 mL/min at 37 °C to flush the coronary vessels with carbogen-bubbled Ca^2+^-free PSS for 5 min. Following this initial perfusion, the hearts underwent retrograde perfusion with the same PSS enriched with Worthington type II collagenase (0.5 mg/mL), 1 mg/mL bovine serum albumin (Sigma), and 10 µmol/L CaCl_2_ for 18–20 min. The perfusate was continuously aerated with carbogen (95% O_2_-5% CO_2_) and maintained at 37 °C. Afterward, the enzymes were washed out using a modified KB medium [6], and the heart was removed from the perfusion system. The ventricles were dissected and gently triturated to release the cells into the KB medium. The resulting cell suspension was filtered and stored in KB medium at 22 °C until use.

### 4.5. RNA Isolation, Sequencing and Analysis

RNA was extracted directly from the cardiomyocytes using TRIzol (Invitrogen, Thermo Fisher Scientific, Inc., Dreieich, Germany) and then subjected to chloroform extraction (Sigma-Aldrich, Schnelldorf, Germany) per the manufacturer’s guidelines. The concentration and purity of the RNA were assessed using a Nanodrop spectrophotometer (Thermo Fisher Scientific, Inc., Dreieich, Germany).

Purification of the isolated RNA was performed using the RNeasy mini kit (Qiagen, Hilden, Germany) according to the manufacturer’s protocol. RNA quantity and quality were evaluated with both Nanodrop (Thermo Fisher Scientific, Inc., Dreieich, Germany) and the Qi-RNA kit (Thermo Fisher Scientific, Inc., Dreieich, Germany). The samples were then prepared utilizing the NEB Ultra II RNA kit (New England Biolabs, Ipswich, MA, USA), following the provided instructions, including the NEBNext Poly(A) magnetic isolation module for mRNA (New England Biolabs, Ipswich, MA, USA) and unique dual-indexing. The concentration, size distribution, and quality of the resulting libraries were analyzed using a Qubit 4 fluorometer (Thermo Fisher Scientific, Inc., Dreieich, Germany) with a high-sensitivity dsDNA kit (Invitrogen, Carlsbad, CA, USA), as well as a 4200 TapeStation with a high-sensitivity D5000 kit (Agilent, Santa Clara, CA, USA). Based on these evaluations, libraries were normalized to their molarity, pooled, and quantified using a library quantification kit designed for Illumina platforms (Roche, Basel, Switzerland) on a StepOnePlus qPCR machine (Thermo Fisher Scientific, Inc., Dreieich, Germany). Ultimately, the pooled libraries were loaded at a concentration of 350 pm with 1% PhiX onto an S2 FlowCell and underwent paired-end sequencing (2 × 150 bp) using a NovaSeq 6000 next-generation sequencer (Illumina, San Diego, CA, USA). The RNA-Seq analysis was performed in triplicate.

The quality of the raw FASTQ sequenced reads was initially examined using FastQC v0.11.5 (accessible at http://www.bioinformatics.babraham.ac.uk/projects/fastqc/ accessed on 4 July 2024) [79]. The sequencing data exhibited high quality, characterized by a high Q30 value and a satisfactory mapping rate, demonstrating a successful sequencing run and reliable base calling. Base quality (Phred scores) across the lengths of the reads in each sample can be found in the Supplementary Materials. Following this quality evaluation, the reads underwent a series of processing steps to enhance quality and alignment. Initially, Trimmomatic v0.36 was utilized to trim reads for quality and remove adapter sequences [80]. The surviving trimmed read pairs were then further processed with Fastp to eliminate poly-G tails and address platform-specific artifacts associated with Novaseq/Nextseq [81]. After these quality-trimming procedures, the reads underwent a second quality assessment using FastQC. Upon completion of both quality control and trimming, the reads were aligned to the rat reference genome (mRatBN7.2) using HISAT2 with default settings [82]. The resulting alignments in SAM format were converted to BAM format and sorted by coordinates using SAMtools v1.3.1 [83]. These sorted alignment files were then processed with HTSeq-count v0.6.1p1 [84], utilizing specific options (-s no -t exon -I gene_id) to generate raw counts for subsequent analysis. The raw counts were then normalized using the TPM (Transcripts per Kilobase Million) method to account for gene length and total read count differences across samples, allowing for a more accurate comparison of gene expression levels across different genes and samples.

### 4.6. Western Blot

The obtained freshly isolated cells were centrifuged at 300 rpm at a temperature of 4 °C. The mass of the cell suspension did not exceed 105 μg.

The cell suspension was then lysed with a solution containing RIPA buffer (Thermo Fisher Scientific, Germany) and protease inhibitors. After processing, the sample was homogenized in a homogenizer Minilys (Bertin Technologies, Montigny-le-Bretonneux, France) and centrifuged for 15 min at 18,000 rpm at 4 °C; then, the supernatant was collected. The resulting samples were stored at −80 °C.

Given the potential for microgravity conditions to alter the expression of commonly used housekeeping genes, we performed RNA-seq analysis to identify an appropriate loading control. Traditional housekeeping genes, including β-actin (ACTB), GAPDH, and β-tubulin (TUBB), showed significant expression differences between control and SMG conditions in our dataset. An analysis of ATP1A1 (Na/K-ATPase α-subunit) revealed stable expression with no significant difference between conditions (TPM: control 27.2 ± 4.9 vs. SMG 23.3 ± 5.4, *p* = 0.89); therefore, it was selected as the housekeeping control for Western blot normalization.

The concentration of total protein in the sample was determined according to Bradford (Bio-Rad Protein Assay Dye Reagent Concentrate, Bio-Rad (Bio-Rad Laboratories, Hercules, CA, USA) [85]. The separation of proteins by mass was performed via electrophoresis in polyacrylamide gel (4% concentrating and 7% separating according to Laemmli) with the addition of a buffer containing 5% β-mercaptoethanol to the samples. Colored protein-containing markers (PageRuler Prestained Protein Ladder 10–170 kDa, Thermo Fisher Scientific, Germany) were applied to two lanes to analyze the mass of the studied channel proteins. Electrophoresis was performed in a vertical position. The initial voltage in the chamber was 70 V; after the generation of the concentrating gel, the voltage was changed to 120 V using an electrode buffer (125 mM TRIS, 960 mM glycine, 0.5% SDS, pH = 8.5).

After that, the proteins from the gel were transferred to a nitrocellulose membrane using a semi-dry transfer (Trans-blot SD Semi-Dry Transfer Cell, Bio-Rad, USA) for one hour using a transfer buffer (25 mM TRIS, 192 mM glycine, 20% methanol). To identify the protein after trans-transfer, Puanceo S dye (0.2% and 3% acetic acid) was used. After that, the membrane was washed with deionized water and then incubated for 1 h in a blocking buffer: TBST (TBS and TWEEN 20 0.1%); 3% dry milk.

Next, primary antibodies to channel proteins, dissolved in TBST buffer and 3% dry milk, were applied to the membrane. Incubation took place for 8 h. The primary antibodies used in the work are listed in Table 1.

Next, the membrane was washed in a TBST solution, replacing the solution for 15, 10 and 5 min. After that, secondary goat antibodies against rabbit antibodies were applied at a dilution of 1:10,000 in TBST solution and 3% dry milk. Incubation took place for 1.5 h at room temperature. Then, the membrane was again washed for 15, 10 and 5 min in TBST solution.

The detection of chemiluminescence was performed using peroxidase and luminol (Stable Peroxide Buffer, Luminol/Enhancer, Thermo Scientific, Germany), fixed images of protein bands were analyzed using a ChemiDoc device (Bio-Rad, USA), and ImageJ version 2.9.0 software was used for the quantitative assessment of protein expression levels.

### 4.7. Mechanical Stretch of the Ventricular Myocytes

The mechanical stimulation method utilized in this study has been previously detailed [6,86,87,88]; here, we focus on specific aspects pertinent to the current context. Following the establishment of whole-cell access with the patch pipette (P), a fire-polished glass stylus (S) was affixed to the membrane [6,86,87,88]. When the stylus was freshly polished and the membrane surface was clean, successful attachment was achieved in approximately 70% of attempts. The stylus was then elevated by 2 µm to avert “scratching” the cell’s lower surface against the coverslip during the stretch phase. A motorized micromanipulator (MP 285, Sutter, Novato, CA, USA; accuracy of 0.2 µm) was employed to systematically increase the distance between S and P by up to 12 µm, with P acting as the fixed point [6,88]. Stretching and releasing the cell could be repeated three to four times with the same cell on average.

Our approach demonstrated that local stretching occurred at the cell surface, while the membrane along the line connecting P and S was stretched as anticipated (with approximately 80% of the total membrane surface remaining unaffected) [6,88]. The impact of mechanical stretching on the sarcomere pattern was captured using a slow-scan CCD camera (Princeton Instruments, Trenton, NJ, USA) and analyzed using MetaMorph version 7.10.5 software (Universal Imaging, West Chester, PA, USA). Before attachment, S and P were positioned 40 µm apart.

Increasing the S-P distance by 4 μm caused approximately a 6% local stretch, while increases of 6 μm, 8 μm, and 10 μm resulted in local stretches of approximately 10%, 14%, and 18%, respectively. This discrepancy is likely due to the reduction in local stretch effects from the cell surface towards the interior of the cell, where the optical focus was set [86,87].

Since mechanical stretching of the cell was restricted to the area between S and P, we did not relate stretch-induced currents to whole-membrane capacitance [6].

### 4.8. Whole-Cell Patch-Clamp

Whole-cell patch-clamp recordings of the late current (I_L_), indicative of I_MGC_ or I_SAC_, were performed using an Axopatch 200B amplifier in conjunction with pClamp 10 software (Molecular Devices, San Jose, CA, USA). The data were filtered at 2 kHz, sampled at a frequency of 5 kHz, and subsequently analyzed using the same software.

The myocytes were super-fused in a small recording chamber (RC-26; Warner Instrument Corp., Brunswick, CT, USA) with a volume of 500 µL, which was positioned on an inverted microscope. Freshly isolated brick-like cardiomyocytes could adhere to the glass bottom in two orientations: edgewise (narrow side) and broadwise (broad side) [87,88]. However, both positions yielded similar responses to stretching, while the response to compression varied depending on the cell orientation [89,90]. For the experiments, we specifically selected cells that adhered edgewise and had similar sizes.

Borosilicate glass patch-clamp electrodes had tip resistances ranging from 1.8 to 2.2 MΩ when filled. Access to the cell was achieved by rupturing the patch subsequent to seal formation. To obtain current–voltage relations (*I/V* curves), a series of 20 pulses, each lasting 140 ms at 1 Hz, were applied, beginning from a holding potential of −45 mV to inactivate tetrodotoxin (TTX)-sensitive Na^+^ currents.

Currents in response to short (5 mV) pulse trains applied at −45 mV were utilized to evaluate membrane capacitance and access resistance [6,91], without compensating for capacitive and leak currents. Measurements typically lasted for approximately 20–25 min, during which time access resistance and capacitive current remained stable. The glass apparatus was adjusted to maintain a consistent 40 µm distance between S and P prior to stretch application to minimize the impact of variations in the size of the stretched membrane. The area of mechanical stretching was likely small and remained unspecified, probably considerably less than 25%. During stretching, membrane capacitance measured the total capacitance rather than that of the stretched membrane; given that mechanical stretching was confined to a small region between S and P, we did not normalize the stretch-induced currents (I_L_) by the whole membrane capacitance [5].

To rigorously rule out the possibility that the stretch-induced current arises from leak or seal rupture, we provide the following supporting evidence: patch-clamp recordings were repeated in cell-attached configuration with stylus-induced stretch (*n* = 6), and seal resistance remained stable at 1.6 ± 0.5 GΩ before and 1.5 ± 0.4 GΩ during stretch. Stretching did not evoke single-channel currents in the cell-attached mode. Access resistance and membrane capacitance remained unchanged (*n* = 9), supporting the ionic nature of the observed current. Stretch-induced currents were fully reversible, whereas leak currents typically are not. Furthermore, NO donors (SNAP and DEA-NO) abolished the stretch-induced current when applied during stretch [70,75]; after washout, the current recovered. NO synthase inhibitors (L-NAME and L-NMMA) and the NO scavenger PTIO also abolished stretch-induced currents [76]. Therefore, the inward current observed during stretching can be confidently attributed to the activation of an ion current rather than to a leakage phenomenon [12].

Membrane currents at the end of the pulse, termed «late currents» (I_L_) were plotted as functions of the respective clamp step potential. The intercept of the resulting *I/V* curve with the voltage axis established the zero-current potential (E_0_), which corresponded to the resting membrane potential of a non-clamped cell (ranging between −70 and −80 mV). Previous findings indicated that the late current at the pulse’s end reflects the cell’s response to mechanical stretching in K^+^_in_/K^+^_out_ solutions [6,78].

To identify the mechanically gated current I_SAC_ or I_MGC_, the differential current was calculated. Differential current values were determined as the difference between control current values (^C^I_L_) and current values under the condition of cell stretch (^S^I_L_), specifically at −80 mV, designated as ΔI_L_ or I_MGC_ [6,78].

### 4.9. Statistics

To assess the TPM of channel genes, there were seven rats (*m* = 7) in the control group from which seven samples of isolated ventricular myocytes (*n* = 7) were taken. To assess TPM in the group of rats subjected to simulated microgravity, four animals (*m* = 4) were taken, from which four samples of isolated ventricular myocytes (*n* = 4) were obtained.

For analysis of the amount of TRPM7 channel protein using Western blotting, 11 rats (*m* = 11) were taken for control experiments, from which 11 samples of isolated cardiomyocyte suspension were obtained (*n* = 11), and for experiments with rats subjected to simulated microgravity, 8 animals (*m* = 8) were taken, from which 8 samples of isolated cardiomyocyte suspension were obtained (*n* = 8). For stretch experiments with the seal resistance in a cell-attached configuration, the number of studied cells *n* = 6; number of rats *m* = 2.

To study the currents determined by the magnitude of cell stretch, the patch clamp experiments were performed on cells (*n* = 29) isolated from 13 rats (*m* = 12). In this case, four rats were taken for the control experiments (*m* = 4), and nine animals were taken for the experiments with rats subjected to SMG (*m* = 9). Note that the value “*n*” refers only to those experiments that were completed to the full end of the protocol. Experiments that were not completed to the end of the protocol were not taken into account.

In each of the three subgroups of SMG-stretched cardiomyocytes, the results (no change, slight increase in current, and slight decrease in current) were obtained from three different rats (*m* = 3), ensuring biological replication across animals.

Data collected using the patch-clamp technique were analyzed with the commercial software package Molecular Devices Axon pClamp 10.2. To evaluate differences within a group of rats, significant variations were assessed using one-way repeated measures analysis of variance (ANOVA for RM), followed by the Holm–Sidak test as a post hoc analysis. Student’s *t*-test was used for intergroup comparisons. A threshold of statistical significance was established at *p* < 0.05. The normality of sample distributions was confirmed using the Shapiro–Wilk test. The results were expressed as mean ± standard error of the mean (SEM), with *n* indicating the number of experiments and *m* representing the number of rat hearts.

Differential gene expression analysis was conducted utilizing the DESeq2 approach within the Galaxy platform (Galaxy Project Team, Penn State University, State College, Pennsylvania). Genes with a *p*-value less than 0.05 were regarded as differentially expressed. The experiments were performed in triplicate, following standard practice, and the results are expressed as mean ± SEM.

The Mann–Whitney test was used to statistically evaluate the difference in protein amounts. The result was considered statistically significant if the *p*-value was less than 0.05 and expressed as mean ± SEM.

## 5. Conclusions

In this study, we show that SMG alters the transcriptional landscape of genes encoding MGCs and MSCs in rat ventricular cardiomyocytes. Although not all changes reached statistical significance, consistent expression trends suggest the biologically meaningful modulation of ion channel regulation. Our data indicate that the I_MGC_ observed under SMG arises from the collective activity of multiple MGCs, rather than a single dominant channel. While TRPM7 may be one of the contributors, the inward rectification and overall biophysical profile of I_MGC_ are more consistent with a composite current mediated by a heterogeneous channel population. The definitive identification of individual channel contributions will require targeted genetic or pharmacological interrogation.

Notably, cardiomyocytes from SMG-exposed animals displayed variable electrophysiological responses to stretch, likely reflecting cell-to-cell differences in protein expression or intracellular signaling dynamics. These heterogeneous responses suggest that SMG triggers adaptive remodeling of the mechanoelectrical interface, possibly as a compensatory mechanism for sustained mechanical unloading.

We recognize the limitations inherent in transcriptomic profiling and the exploratory nature of this investigation. Nonetheless, our findings lay important groundwork for future research aimed at delineating the molecular mediators of cardiac adaptation to altered gravity. Such insights may prove essential in protecting cardiovascular function during extended spaceflight and addressing cardiac deconditioning in immobilized patients on Earth.

## Figures and Tables

**Table 1:** Antibodies used for the identification of channel proteins by Western blot.

Antibodies	Dilution	Origin	Country of Origin
TRPM 7	1:1000	Rabbit	ABclonal, San Diego, CA, USA
α-subunit of Na/K-ATPase	1:100,000	Rabbit	Abcam Limited, Cambridge, UK

## Data Availability

The data that support the findings of this study are available in Gene Expression Omnibus (GEO) at https://www.ncbi.nlm.nih.gov/geo/query/acc.cgi?acc=GSE285899, accessed on 16 July 2024. All further data gathered in this study (including manual patch-clamp measurements and their analysis procedures) are available from the corresponding author upon request.

## Supplementary Materials

The following supporting information can be downloaded at: https://www.mdpi.com/article/10.3390/ijms26146653/s1.

## Abbreviations

AC	Adenylate Cyclase
ANOVA	Analysis of Variance
AP	Action Potential
APD	Action Potential Duration
ATP	Adenosine Triphosphate
BAPTA	1,2-bis(o-aminophenoxy)ethane-N,N,N′,N′-tetraacetic acid
Ca^2+^	Calcium
EGTA	Ethylene Glycol Tetraacetic Acid
ER	Endoplasmic Reticulum
ESC	Embryonic Stem Cell
FASTQ	Text-based format for storing nucleotide sequences and quality scores
FASTQC	Quality Control tool for High-Throughput Sequence Data
HEK	Human Embryonic Kidney
HEPES	4-(2-hydroxyethyl)-1-piperazineethanesulfonic acid
HISAT	Hierarchical Indexing for Spliced Alignment of Transcripts
IK	Delayed Rectifier Potassium Current
I_L_	Late Membrane Current
I_MGC_	Mechanically Gated Current
I_SAC_	Stretch-Activated Current
K_ATP_	ATP-sensitive Potassium Channel
KB	Kraftbrühe
KOH	Potassium Hydroxide
K_V_	Voltage-Gated Potassium Channel
MEF	Mechanoelectrical Feedback
MG	Microgravity
MGC	Mechanically Gated Channel
NFAT	Nuclear Factor of Activated T-cells
NMDA	N-Methyl-D-Aspartate
NO	Nitric Oxide
NOS	Nitric Oxide Synthase
PDE	Phosphodiesterase
PSS	Physiological Salt Solution
RNA	Ribonucleic Acid
SD	Standard Deviation
SEM	Standard Error of the Mean
SMG	Simulated Microgravity
TASK	TWIK-related Acid-sensitive K+ channel
TBST	Tris-Buffered Saline with Tween 20
TPM	Transcripts Per Kilobase Million
TREK	TWIK-Related K+ Channel
TRIS	Tris(hydroxymethyl)aminomethane
TRP	Transient Receptor Potential
TTX	Tetrodotoxin
