# Supplementary material for: Simulated Microgravity Attenuates Stretch Sensitivity of Mechanically Gated Channels in Rat Ventricular Myocytes
Source: Int J Mol Sci. 2025 Jul 11;26(14):6653. doi: 10.3390/ijms26146653 (PMC12294224; doi:10.3390/ijms26146653)
Supplement: Supplementary file 1 [file ijms-26-06653-s001.zip › ijms-3683997-supplementary.pdf]

Supplementary Table S1:

Channel types, **mechanosensitivity classification**, and **justification for inclusion**.

Channel	Type	Mechanosensitivity Status	Justification for inclusion
Kir6.2	MSC (K_{ATP})	Established mechanosensitive	↑ TPM in SMG
Kir2.1	MSC	Modulated by tension/lipids	↑ TPM; role in RMP
Kv1.5	VGC	Limited evidence	↓ TPM; relevance to atrial current
Kv7.2	VGC	Possible indirect modulation	↑ TPM; IKs contributor
